# Inhibition of AIM2 inflammasome-mediated pyroptosis by Andrographolide contributes to amelioration of radiation-induced lung inflammation and fibrosis

**DOI:** 10.1038/s41419-019-2195-8

**Published:** 2019-12-20

**Authors:** Jian Gao, Shuang Peng, Xinni Shan, Guoliang Deng, Lihong Shen, Jian Sun, Chunhong Jiang, Xiaoling Yang, Zhigang Chang, Xinchen Sun, Fude Feng, Lingdong Kong, Yanhong Gu, Wenjie Guo, Qiang Xu, Yang Sun

**Affiliations:** 1 State Key Laboratory of Pharmaceutical Biotechnology, Deparment of Biotechnology and Pharmaceutical Sciences, School of Life Sciences, Nanjing University, 163 Xianlin Avenue, Nanjing, 210023 China; 2 Key Laboratory of High Performance Polymer Materials and Technology of Ministry of Education, Department of Polymer Science & Engineering, School of Chemistry & Chemical Engineering, Nanjing University, Nanjing, 210023 China; 3 State Key Laboratory of Innovative Nature Medicine and TCM Injections, Jiangxi Qingfeng Pharmaceutical Co., Ltd, Ganzhou, China; 4 The First Affiliated Hospital of Nanjing Medical University, Nanjing, 210029 China; 5 Jiangsu Key Laboratory of New Drug Research and Clinical Pharmacy, Xuzhou Medical University, 209 Tongshan Road, Xuzhou, 221004 Jiangsu China; 6 Chemistry and Biomedicine Innovation Center (ChemBIC), Nanjing University, Nanjing, 210023 China

**Keywords:** Pharmacology, Respiratory tract diseases

## Abstract

Radiation-induced lung injury (RILI) is one of the most common and fatal complications of thoracic radiotherapy, whereas no effective interventions are available. Andrographolide, an active component extracted from *Andrographis paniculate*, is prescribed as a treatment for upper respiratory tract infection. Here we report the potential radioprotective effect and mechanism of Andrographolide on RILI. C57BL/6 mice were exposed to 18 Gy of whole thorax irradiation, followed by intraperitoneal injection of Andrographolide every other day for 4 weeks. Andrographolide significantly ameliorated radiation-induced lung tissue damage, inflammatory cell infiltration, and pro-inflammatory cytokine release in the early phase and progressive fibrosis in the late phase. Moreover, Andrographolide markedly hampered radiation-induced activation of the AIM2 inflammasome and pyroptosis in vivo. Furthermore, bone marrow-derived macrophages (BMDMs) were exposed to 8 Gy of X-ray radiation in vitro and Andrographolide significantly inhibited AIM2 inflammasome mediated-pyroptosis in BMDMs. Mechanistically, Andrographolide effectively prevented AIM2 from translocating into the nucleus to sense DNA damage induced by radiation or chemotherapeutic agents in BMDMs. Taken together, Andrographolide ameliorates RILI by suppressing AIM2 inflammasome mediated-pyroptosis in macrophage, identifying Andrographolide as a novel potential protective agent for RILI.

## Introduction

Radiotherapy is an important therapeutic modality for a variety of tumors such as lung cancer, breast cancer, prostate cancer, and renal cell carcinoma^1–4^. Despite significant improvements in radiotherapy delivery methods, radiation-induced lung injury (RILI) is still the most common and severe complication in the treatment of thoracic tumors, which not only limits the further application of fractionated radiotherapy but also results in a poor prognosis for patients. The development of RILI is mainly divided into radiation pneumonitis in the early phase and the later radiation fibrosis in the late stage. An acute inflammatory reaction occurs within a few weeks of radiation and is characterized by the release of pro-inflammatory cytokines and the accumulation of immune cells in lung tissues, while chronic fibrosis presents months to years later and eventually leads to permanent impairment of lung function^5–8^. Currently, the pathogenesis of RILI remains unclear and there are no effective interventions in the clinic. Therefore, novel strategies that can prevent or reverse RILI are urgently needed.

Accumulating evidence suggests that AIM2 inflammasome-mediated pyroptosis both in macrophages and epithelial cells plays a critical role in the development of radiation-induced tissue injury^9,10^. Pyroptosis, recognized as a form of cell death distinct from apoptosis, is caused by activation of inflammasomes^11^. The AIM2 inflammasome, comprised of AIM2 and ASC, recognizes double-stranded DNA breaks (DSBs) caused by ionizing radiation and chemotherapeutic agents^12,13^. Under radiation, AIM2 translocates into the nucleus and localizes at the foci of DSB, forming specks and recruits ASC, which leads to Caspase-1 activation. Activated Caspase-1 then causes the enzymolysis of the inflammatory cytokines interleukin (IL)-1β and IL-18 or the cleavage of Gasdermin D, which induces pore formation on the plasma membrane, resulting in cell swelling and the release of cytosolic contents such as lactate dehydrogenase (LDH) and pro-inflammatory cytokines^14–18^.

Andrographolide, an active component extracted and purified from *Andrographis paniculate*, is known to have various pharmacological effects, such as antibacterial, anti-inflammatory, and anticancer activities^19–23^. In a previous study, we reported that Andrographolide could alleviate lipopolysaccharide-induced lung injury^24^. Andrographolide is prescribed as a treatment for inflammatory diseases, including rheumatoid arthritis, asthma, laryngitis, and especially upper respiratory tract infection, in China and southeast Asia countries^25–28^. Therefore, we hypothesized that Andrographolide might be a potent therapeutic strategy in the prevention of radiation-induced pneumonitis and fibrosis after radiotherapy for thoracic tumors.

To investigate this hypothesis, we designed a mouse model of RILI in which mice were exposed to 18 Gy of whole-thorax irradiation. Andrographolide was delivered at a dose of 5, 10, or 20 mg/kg starting 24 h post-irradiation for a duration of 4 weeks. Our results demonstrated that Andrographolide significantly attenuated the release of pro-inflammatory cytokines, infiltration of immune cells, and lung damage in acute pneumonitis as well as collagen deposition during the development of radiation fibrosis, thus protecting mice and prolonging survival of RILI. Using primary cultured bone marrow-derived macrophage (BMDM) radiation model, we revealed that Andrographolide markedly hampered the activation of the AIM2 inflammasome and pyroptosis in macrophages by preventing AIM2 from translocating into the nucleus to sense DNA damage induced by radiation. Thus Andrographolide alleviates RILI by suppressing AIM2 inflammasome-mediated pyroptosis in macrophages.

## Materials and methods

### Mice

Female C57BL/6 mice (6–8 weeks old, 18–22 g) were purchased from the Model Animal Research Center of Nanjing University, Nanjing, China. *Nlrp3*^−/−^ mouse was a gift from Professor Rongbin Zhou (University of Science and Technology of China). Mice were housed under standard laboratory conditions (22 ± 2 °C, 55 ± 10% humidity, and 12–12-h light–dark cycle), and sterilized food and water were supplied. Animal welfare and experimental procedures were carried out strictly in accordance with the Guide for the Care and Use of Laboratory Animals (National Institutes of Health, USA) and the related ethical regulations of our university. All efforts were made to minimize animals’ suffering and to reduce the number of animals used.

### Reagents and antibodies

Andrographolide (Andro, Cat # 365645, chemical structure is shown in Supplementary Fig. S1a) was purchased from Sigma-Aldrich (St. Louis, MO). Enzyme-linked immunosorbent assay (ELISA) Kits for tumor necrosis factor (TNF)-α, IL-6 and IL-1β were purchased from Dakewe Biotech Co. Ltd (Shenzhen, China). Anti-Caspase-1 (Cat # ab108362), anti-Collagen I (Cat # ab34710), and anti-α-SMA (Cat # ab124964) were purchased from Abcam (Cambridge, USA). Anti-Gasdermin D (Cat # sc-81868), anti-ASC (Cat # sc-271054), and anti-AIM2 (Cat # sc-293174, sc-137971) were purchased from Santa Cruz Biotechnology (Santa Cruz, CA). Anti-p-Histone-H2A.X (Ser139) (Cat # ZRB05636) was purchased from Sigma-Aldrich. Anti-Histone H3 (Cat # 4499), anti-p-p65 (Cat # 3033), anti-p65 (Cat # 8242), anti-p-ERK (Cat # 4370), anti-ERK (Cat # 9102), anti-p-JNK (Cat # 4668), anti-JNK (Cat # 9252), anti-p-p38 (Cat # 4511), and anti-p38 (Cat # 8690) were purchased from Cell Signaling Technology (Beverly, MA). Anti-GAPDH (Cat # M20006) and anti-β-Actin (Cat # M20011) was purchased from Abmart (Shanghai, China). Anti-mouse Gr1-PE (clone RB6-8C5, Cat # 12-5931-81), CD3-APC (clone 145-2C11, Cat # 17-0031-81), and CD11b-FITC (clone M1/70, Cat # 11-0112-82) antibodies were purchased from Thermo Scientific (Waltham, MA). Armenian Hamster IgG Isotype Control APC (Cat # 17-4888-82), Rat IgG2b kappa Isotype Control PE (Cat # 12-4031-82), and Rat IgG2b kappa Isotype Control FITC (Cat # 11-4031-82) were purchased from Thermo Scientific (Waltham, MA). Alexa Fluor 488 Goat anti-mouse IgG (H+L) Secondary Antibody (Cat # A11001) and Alexa Fluor 594 Goat anti-Rabbit IgG (H+L) Secondary Antibody (Cat # A11037) were purchased from ThermoFisher Scientific (Waltham, MA). The GTVision^TM^ Anti-mouse/Anti-rabbit Immunohistochemical Analysis Kit (Cat # GK500705) was purchased from GeneTech Company (Shanghai, China). All other chemicals were obtained from Sigma-Aldrich (St. Louis, MO).

### BMDM isolation and cell culture

Bone marrow cells were flushed out from the femurs and tibias of female C57BL/6 mice. After centrifugation for 5 min at 300 × *g*, erythrocytes were eliminated and the remaining cells were cultured with Dulbecco’s modified Eagle’s medium supplemented with 10% fetal bovine serum (FBS) and 20 ng/mL macrophages colony-stimulating factor (M-CSF; Peprotech, Rock Hill, NJ, Cat # 315-02). Culture fluid was exchanged with culture medium every 3 days. Under these conditions, adherent macrophages were obtained within 6–7 days. Cells were harvested and seeded on 24-well plates. After culture for 6 h without M-CSF, the cells were used for the experiments as BMDMs^29^.

Human monocytic THP-1 cell line obtained from Shanghai Institute of Cell Biology (Shanghai, China) THP1 cells were cultured in RPMI 1640 (GIBCO, Grand Island, NY) containing 10% FBS (GIBCO, Grand Island, NY) in 5% CO_2_ at 37 °C. Before further treatment, THP-1 cells were treated with phorbol myristate acetate (500 nM) for 3 h and then exchanged with fresh medium to culture overnight.

### Irradiation experiments in vivo and in vitro

BMDMs were exposed to X-ray radiation to attain a dose of 8 Gy at a dose rate of 297.43 cGy/min at Irradiation Center of the First Affiliated Hospital, Nanjing, China. Mice were anesthetized and exposed to whole-thorax radiation by timed exposure to X-ray resources at a dose rate of 200.00 cGy/min and a cumulative radiation dose of 18 Gy at Irradiation Center of the People’s Hospital of Jiangsu Province, Nanjing, China. Mice were randomly divided into five groups before irradiation, including the irradiated group (IR), the groups treated with irradiation followed by three different doses of Andrographolide, and a vehicle control group (*n* = 60 in each group). Andrographolide sulfonate (Xi-Yan-Ping, Z20026249 approved by Jiangxi Qingfeng Pharmaceutical Co., Ltd) was diluted with phosphate-buffered saline (PBS) and administered by intraperitoneal injection every other day at doses of 5, 10, and 20 mg/kg from 24 h to 4 weeks post-irradiation. Control and IR group were intraperitoneally injected with PBS.

### Sample collection

Twelve mice from each group were anesthetized at 4, 8, 20, and 30 weeks after irradiation. And then six mice were sacrificed for collecting 500 μL blood. Mice chest cavity was opened and the trachea was exposed carefully. A small incision was made at the top of trachea via a needle to insert an 18-gauge catheter and secured tightly with 3-0 silk suture. Bronchoalveolar lavage (BAL) was performed by perfusing 0.5 mL PBS for three times. The recovery rate of the fluid was about 80%. The serum and the supernatant in BAL fluid (BALF) were collected and used for ELISAs. The pellet from the BALF was suspended and then used to analyze the inflammatory cells by flow cytometry. Wet weight of the lung tissue from another six mice was recorded and the lung tissue was separated into several parts for histology, reverse transcriptase polymerase chain reaction (PCR), western blot, and immunohistochemical analysis. The last 12 mice were observed for survival analysis. Data collection and analysis was performed blindly; the experimenters were unaware of the group assignment and animal treatment.

### Flow cytometric analysis

Cells from BALF were washed with cold PBS and stained with specific antibodies for 30 min at 4 °C in dark and analyzed on BD FACSCalibur (BD Bioscience, CA).

### Histological analysis and immunohistochemistry

Removed lungs were immersed in 4% paraformaldehyde for 48 h and embedded in paraffin. Histopathological study was made using hematoxylin & eosin stain. Alveolar congestion, hemorrhage, infiltration, or aggregation of inflammatory cells in airspaces or vessel walls and the thickness of the alveolar walls were assessed by 0–4 point semi-quantitative histological analysis (4: Extremely serious, 3: Serious, 2: Middle, 1: slight, 0: Normal). Histology score is an average of all the scores. Masson’s trichrome stain was used to detect the collagen deposition. Collagen volume fraction was performed by the ImageJ software (https://imagej.nih.gov/ij/). Stained images were converted to RGB stack type (Image-Type-RGB stack), adjusted threshold (Image-Adjust-Threshold), and then selected measurements as area fraction to analyze. Acquired data are expressed as a histogram of mean ± S.E.M. of five fields per mice in every group. For immunohistochemistry, paraffin-embedded lung sections were heat-fixed, deparaffinized, rehydrated, antigen retrieved, blocked with 3% goat serum, and incubated with the indicated antibodies overnight at 4 °C, and then the slides were detected using the GTVision^TM^ Anti-mouse/Anti-rabbit Immunohistochemical Analysis Kit (GeneTech, Shanghai, China) according to the manufacturer’s instructions. The quantification of immunohistochemistry was performed using Plugins called IHC Profiler (https://sourceforge.net/projects/ihcprofiler). The score was divided into four levels (4: High positive, 3: Positive, 2: Low positive, 1: Negative). Acquired data are expressed as a histogram of mean ± S.E.M. of five fields per mice in every group.

### Quantitative real-time PCR (qPCR) analysis

qPCR was performed as previously described^30^. Total RNA were extracted from cells or tissues and reverse transcribed to cDNA and subjected to qPCR, which was performed with the BioRad CFX96 Touch^TM^ Real-Time PCR Detection System (BioRad, CA) and iQ SYBR Green Supermix (Bio-Rad, Cat # 1708882), and threshold cycle numbers were obtained using the BioRad CFX manager software version 5.0. The program for amplification was 1 cycle of 95 °C for 2 min followed by 40 cycles of 95 °C for 15 s and 60 °C for 30 s. The primer sequences used in this study are listed in Supplementary Table 1.

### Western blot analysis

Protein was extracted in lysis buffer (30 mmol/L Tris, pH 7.5, 150 mmol/L sodium chloride, 1 mmol/L phenylmethylsulfonyl fluoride, 1 mmol/L sodium orthovanadate, 1% Nonidet P-40, 10% glycerol, and phosphatase and protease inhibitors). The protein content of the supernatant was determined using a BCA Protein Assay Kit (Pierce, Rockford, IL, USA) and then fractionated by sodium dodecyl sulfate-polyacrylamide gel electrophoresis (SDS-PAGE) before being electrophoretically transferred onto polyvinylidene fluoride membranes (Millipore Corp., Bedford, MA, USA). The membrane was blocked with 5% nonfat milk for 1 h at room temperature and then incubated with the indicated primary antibodies overnight at 4 °C and incubated with horseradish peroxidase-coupled secondary antibody. Detection was performed using a LumiGLO chemiluminescent substrate system (KPL, Gaithersburg, MD).

### LDH release assay and ELISA

LDH activity was measured by using a CytoTox 96® Nonradioactive Cytotoxicity Assay Kit (Promega, G1780). Cytokines in serum, BALF supernatant, and supernatant harvested from BMDM culture were detected by ELISA according to the manufacturer’s instructions. Optical density value was determined at 450 nm.

### Immunofluorescence

The lung tissue sections or fixed cells were blocked in Blocking buffer (1 × PBS, 5% anti-goat serum, 0.01% Triton X-100) for 1 h and then incubated with the primary antibodies (1:50–1:100) at 4 °C overnight. Samples were then washed with PBS 3 times and stained with fluorophore-conjugated secondary antibodies (1:500) at room temperature for 90 min. After a second wash, samples were observed with a confocal laser scanning microscope (Olympus, Lake Success, NY).

### FAM-FLICA Caspase-1 assay

Activated Caspase-1 was detected by the FAM-FLICA Caspase-1 Assay Kit (ImmunoChemistry Technologies, Cat # 98) according to the manufacturer’s instructions. Briefly, for flow cytometric analysis, 290 μL cells were incubated with 10 μL 30× diluted FLICA staining solution (50 μL DMSO FLICA stock 1:5 in PBS) at 37 °C for 1 h protected from light. After washing with 1× Apoptosis Wash Buffer and staining with propidium iodide (PI; 0.5% v/v), the samples were analyzed by BD FACSCalibur (BD Bioscience, CA).

For tissue sections, cryostat sections (4 μm) were prepared and fixed with acetone for 1 min. The slides were washed with PBST 3 times for 5 min, blocked for 1 h with blocking buffer (1× PBS, 5% bovine serum albumin, 0.2% Tween), and incubated with tissue section staining solution (150× FLICA stock 1:50 in PBS) for 2 h protected from light. After washing with PBST 3 times for 10 min, the slides were set in 1× Apoptosis Wash Buffer and stained with 4,6-diamidino-2-phenylindole. Then samples were observed with a confocal laser scanning microscope.

### Co-immunoprecipitation assay

Proteins from cells were incubated with 2 μg of appropriate antibody at 4 °C overnight and precipitated with protein A/G-agarose beads (Santa Cruz, CA, Cat # sc-2003) for another 4 h at 4 °C. The beads were washed with lysis buffer 5 times by centrifugation at 1000 × *g* for 5 min at 4 °C. The immunoprecipitated proteins were separated by SDS-PAGE and western blotting was performed with the indicated antibodies.

### RNA interference

The small interfering RNA (siRNA) duplexes for negative control and AIM2 were designed by GenePharma (Shanghai, China). THP-1-derived macrophages were electrotransfected with siRNA of negative control (5′-UUCUCCGAACGUGUCACGUTT-3′) and AIM2 (5′-GCCUGGAUAACAUCACUGATT-3′) by using BTX Gemini Twin Wave Electroporation System (Harvard Bioscience, MA) according to the manufacturer’s instructions and then cultured for 24 h.

### Statistical analysis

All experiments are randomized and blinded. Block randomization was used to randomize samples/mice into groups of similar sample size. No samples and animals were excluded from analysis. All experiments were performed at least in triplicates. Statistical analysis was performed with the GraphPad Prism 5.0 software (San Diego, CA, USA). Error bars depict standard error of mean (S.E.M.) of each experiment. One-way analysis of variance followed by Dunnett’s post hoc test was used to evaluate the differences between various experimental and control groups when there were more than two groups. Post tests were run only if *F* achieved *P* < 0.05. Student’s *t* test was used to determine the significance of difference between two groups. *P* values <0.05 were considered statistically significant. Statistical power analysis was used to ensure adequate sample size for detecting significant difference between samples. The variance is similar between groups that are being statistically compared.

## Results

### Andrographolide protected mice from lung injury induced by thoracic radiotherapy

**Fig. 1:**
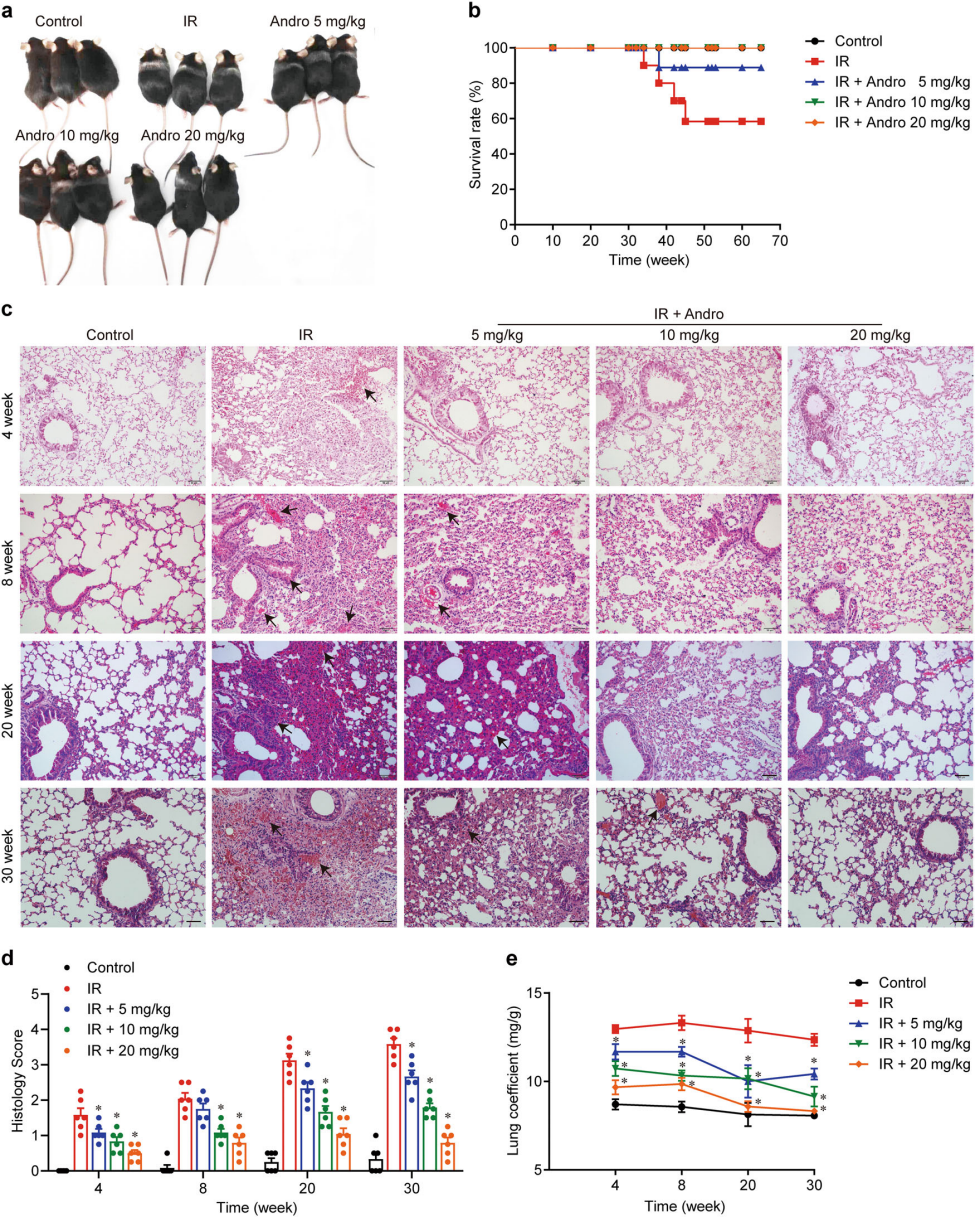
Andrographolide protected mice from radiation-induced lung injury. Mice were exposed to 18 Gy irradiation and then treated with the indicated doses of Andrographolide for 4 weeks after irradiation. **a** Representative images of mice at 8 weeks post-irradiation. **b** Kaplan–Meier survival analysis of mice in different groups, *n* = 12 per group. **c** Representative images of H&E staining of lung tissues from each group. Alveolar hemorrhage and the thickness of the alveolar walls were indicated by arrows. Scale bar, 50 μm. **d** Semi-quantitative histological analysis of lung tissues of five fields per mouse in every group. **e** Lung coefficient of mice in each group. The results are shown as the mean ± S.E.M., *n* = 6 mice per group. *P < 0.05 vs the IR group.

We designed a mouse model of RILI in which mice were exposed to 18 Gy of whole-thorax irradiation. Andrographolide (Supplementary Fig. S1a) was administered at a dose of 5, 10, or 20 mg/kg starting 24 h post-irradiation for a duration of 4 weeks (Supplementary Fig. S1b). Mice exhibited apparent hair loss and discoloration in the area of exposure at 8 weeks post-radiation (Fig. 1a) and death, whereas mice treated with Andrographolide were protected from this damage and showed significant improvement in survival (Fig. 1b). In accordance with this observation, lung tissues in the radiation group displayed obvious pulmonary edema; congestion; and progressive thickened alveolar walls, collapsed alveoli, and collagen deposition from 4 to 30 weeks post-irradiation, while Andrographolide treatment markedly mitigated these inflammatory pathological changes (Fig. 1c, d). In addition, the lung coefficient (lung weight/body weight), which mainly reflects the degree of pulmonary edema, was significantly reduced by Andrographolide treatment (Fig. 1e).

### Andrographolide attenuated inflammatory cell infiltration and decreased the expression of pro-inflammatory cytokines in the lung

**Fig. 2:**
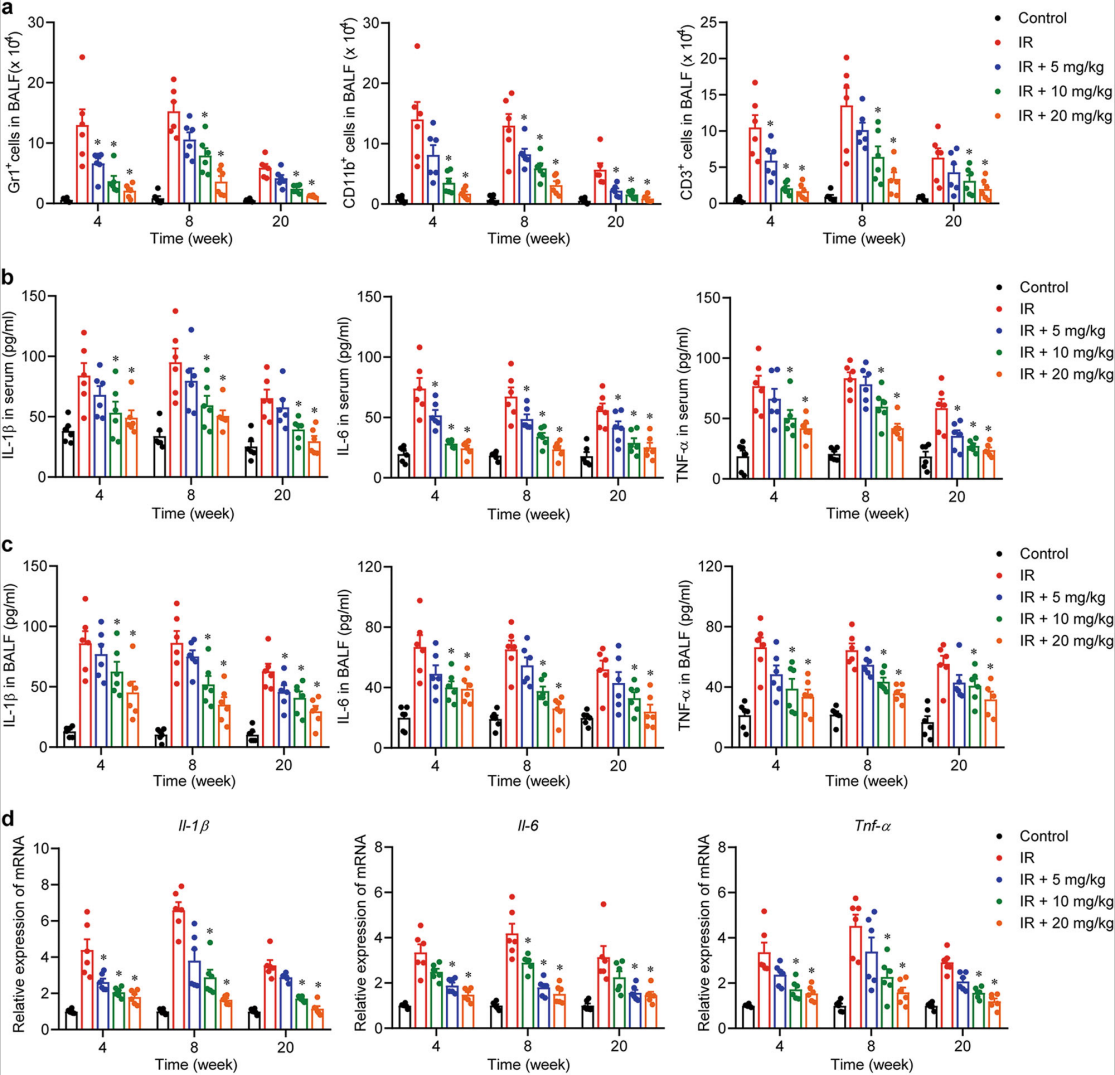
Andrographolide ameliorated radiation-induced pneumonitis in the early phase. **a** Cells in BALF were stained with Gr1-PE, CD11b-FITC, and CD3-APC and then analyzed by flow cytometry. Cytokines in serum (**b**) and BALF (**c**) from each group were detected by ELISA. **d** Relative mRNA expression of IL-1β, IL-6, and TNF-α was measured by qPCR. Data are expressed as the mean ± S.E.M., *n* = 6 mice per group, *P < 0.05 vs the IR group.

Mice in the radiation group showed high levels of immune cell infiltration into lung tissues (Supplementary Fig. S2), shown as macrophages (CD11b^+^), neutrophils (Gr1^+^), and T lymphocytes (CD3^+^) (Supplementary Figure S3, Fig. 2a), indicating an acute inflammatory response at 4 and 8 weeks post-radiation. The concentration of representative cytokines in the plasma and BALF, such as IL-1β, IL-6, and TNF-α, were significantly elevated owing to the radiation (Fig. 2b, c). Similarly, the mRNA expression of these cytokines was increased after radiation (Fig. 2d). Collectively, Andrographolide administration significantly attenuated radiation-induced pneumonitis in a dose-dependent manner in the early phase (Fig. 2).

### Andrographolide suppressed radiation-induced epithelial–mesenchymal transition and chronic fibrosis in the lung

**Fig. 3:**
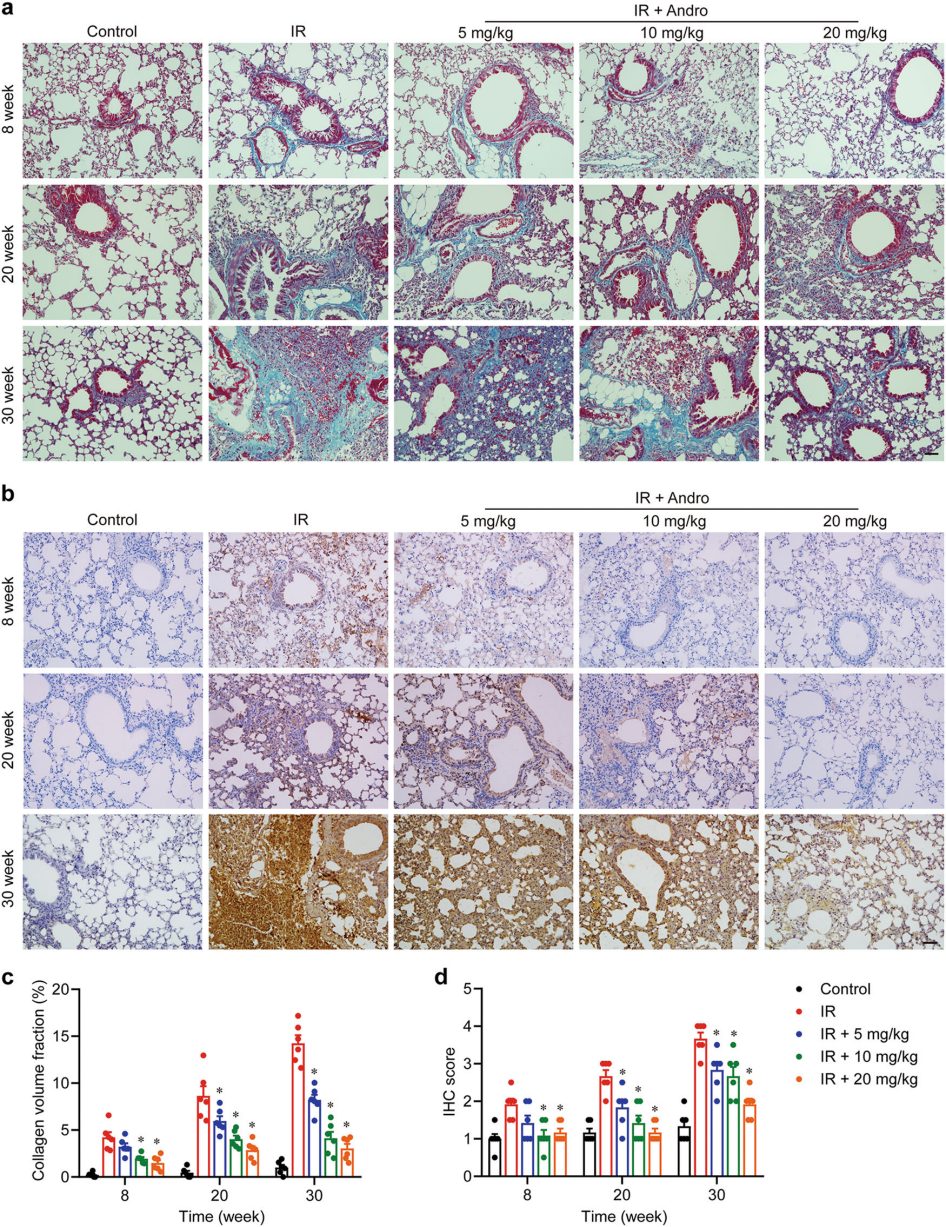
Andrographolide mitigated chronic fibrosis resulting from radiation. **a** Representative images of Masson’s trichrome staining. **b** Representative images of immunohistochemistry staining for collagen I. **c** Quantification of Masson’s trichrome staining. **d** Quantification of collagen I deposition. Scale bar, 50 μm. Data are shown as the means ± S.E.M. of five fields of view per mouse in every group, *n* = 6 mice per group. *P < 0.05 vs the IR group.

**Fig. 4:**
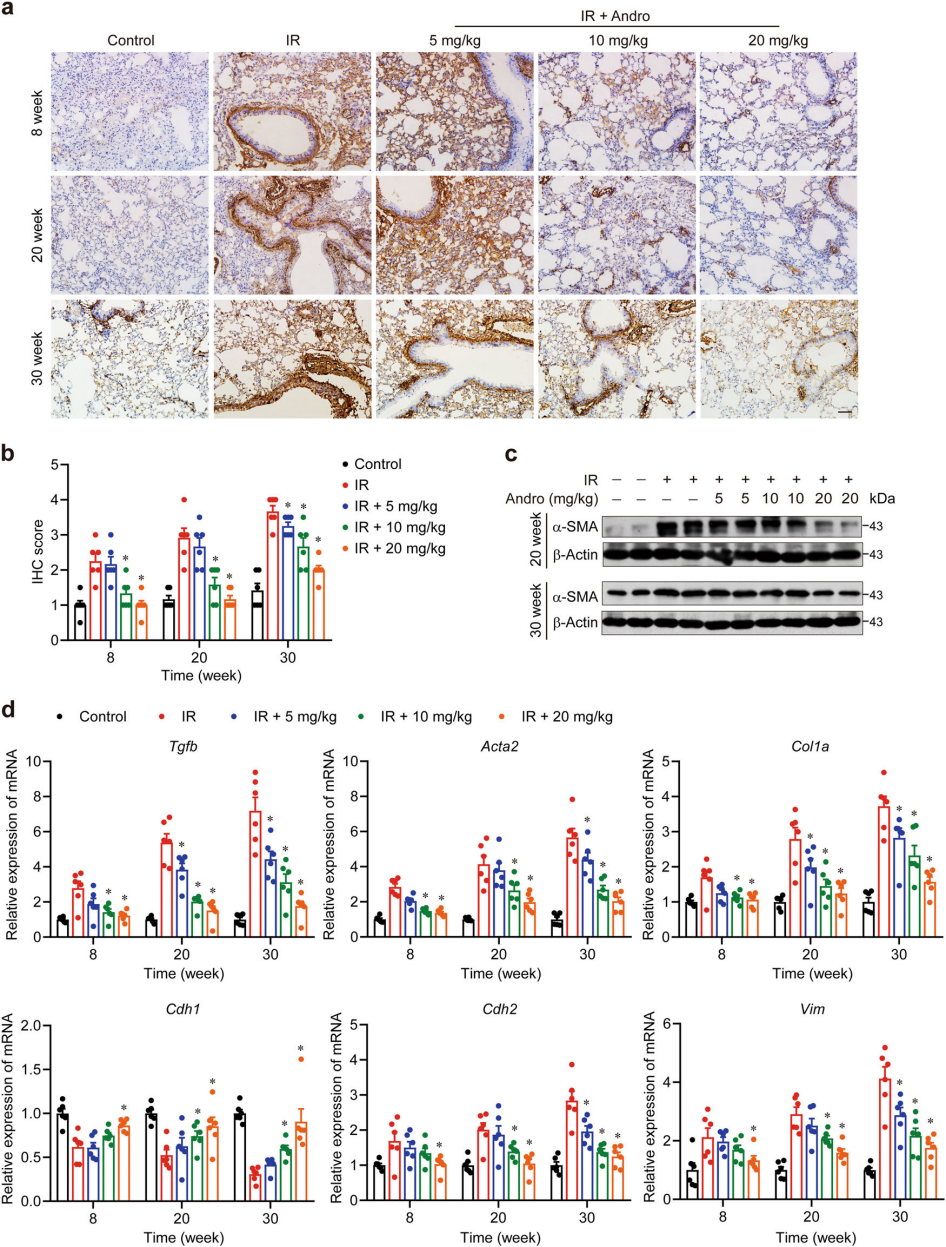
Andrographolide reversed radiation-induced epithelial–mesenchymal transition. **a** Paraffin-embedded lung tissue sections from each group were stained for α-SMA. Scale bar, 50 μm. **b** Quantification analysis of α-SMA-positive cells of lung tissues was shown as the mean ± S.E.M. of five fields per mouse in every group. **c** Western blot analysis of α-SMA in lung tissues from different groups. **d** Relative mRNA expression of TGF-β, α-SMA, Collagen I, E-cadherin, N-cadherin, and Vimentin was examined by qPCR. Data are expressed as the mean ± S.E.M., *n* = 6 mice per group. *P < 0.05 vs the IR group.

Next, we investigated whether Andrographolide could prevent the development of chronic fibrosis in the late phase after radiation. This was confirmed by Masson’s trichrome staining (Fig. 3a, c) and the immunohistochemistry staining for Collagen I (Fig. 3b, d), in which severe deposition of collagen in the lung tissue from the radiation group was observed. We detected the protein level of alpha-smooth muscle actin (α-SMA) and the mRNA expression of transforming growth factor (TGF)-β, α-SMA, Collagen I, E-cadherin, N-cadherin, and Vimentin and found that there was no obvious epithelial–mesenchymal transition, the key event for fibrosis, at 4 weeks after radiation (data not shown). However, the level of α-SMA (Fig. 4a–c) and the expression of TGF-β (*Tgfb*), α-SMA (*Acta2*), Collagen I (*Col1a*), N-cadherin (*Cdh2*), and Vimentin (*Vim*) were markedly upregulated at 8 weeks post-radiation, especially after 20 weeks (Fig. 4d). In contrast, the expression of E-cadherin (*Cdh1*) was significantly decreased, implying that chronic fibrosis was induced by radiation. Notably, Andrographolide treatment dose-dependently blocked radiation-induced epithelial–mesenchymal transition and ameliorated chronic fibrosis in the lung. These results suggest that Andrographolide exerts potent inhibitory effect on both radiation-induced pneumonia in the early phase and pulmonary fibrosis in the late phase.

### Andrographolide attenuated radiation-induced cell death and Caspase-1 activation in lung tissues

**Fig. 5:**
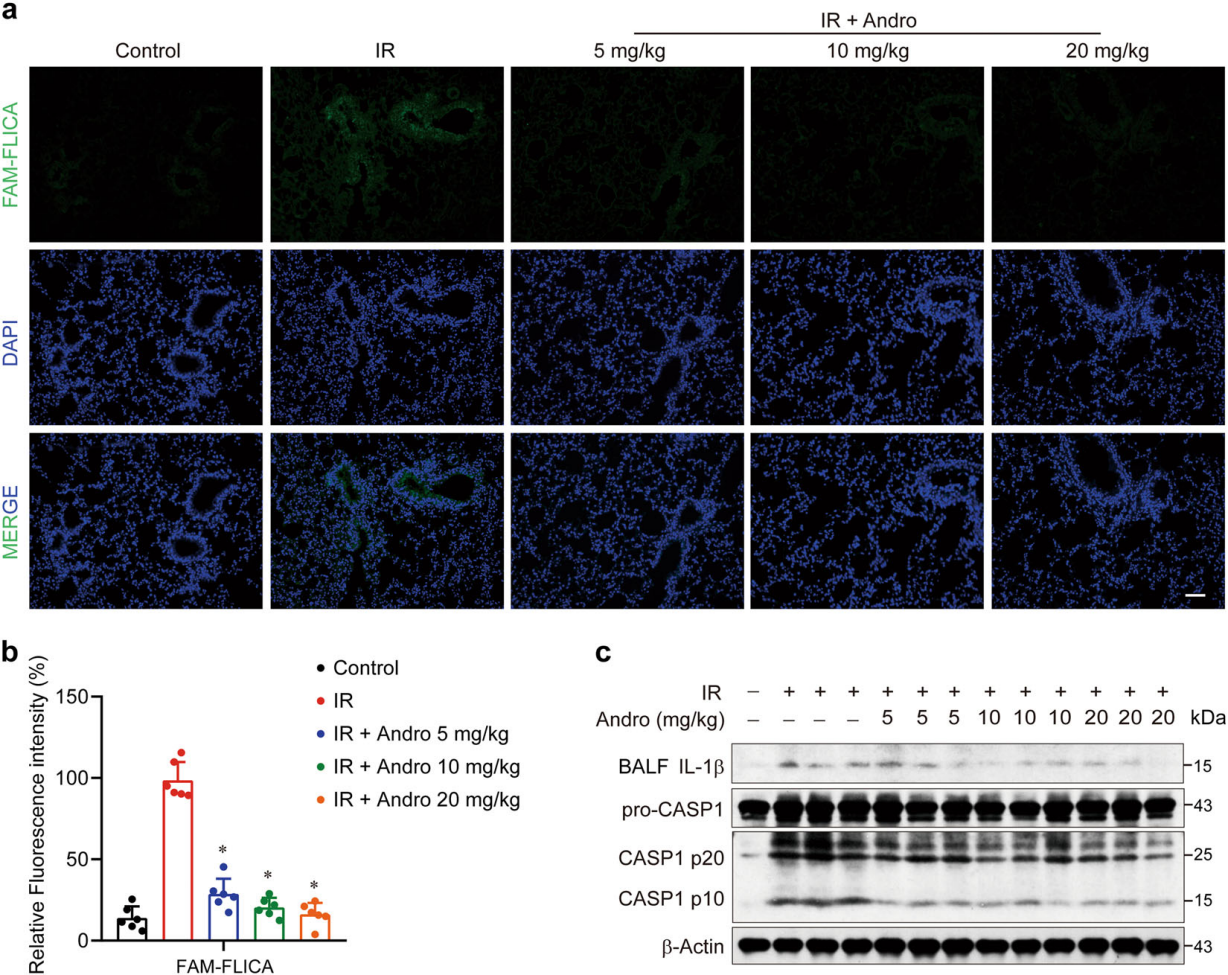
Andrographolide attenuated radiation-induced cell death and Caspase-1 activation in lung tissues. **a** Representative images of Caspase-1 (stained with FAM-FLICA, shown in green) were analyzed via confocal microscopy. Cell nuclei were visualized by DAPI (blue). Scale bar, 50 μm. **b** Fluorescence of FAM-FLICA of lung tissues was quantified (five fields per mouse in every group). **c** Protein was extracted from lung tissues of each group and the expression of Caspase-1 was examined by western blot analysis. Data are expressed as the mean ± S.E.M., *n* = 6 mice per group, *P < 0.05 vs the IR group.

Radiotherapy functions by damaging the double-stranded DNA (dsDNA) and inducing cell death, which was also observed in the injured lung of our mouse model, shown as TUNEL (terminal deoxynucleotidyl transferase-mediated dUTP-fluorescein nick end labeling)-positive cells. Andrographolide apparently suppressed radiation-induced cell death in the lung tissues (Supplementary Fig. S4). However, different forms of cell death, including pyroptosis, apoptosis, and mitotic catastrophe, have been implicated in radiation-induced tissue damage^31–33^. Importantly, activation of Caspase-1 was observed in the lung tissues of mice exposed to radiation by FAM-FLICA Caspase-1 staining (Fig. 5a, b). Similar result was obtained using immunoblotting (Fig. 5c). Taken together, macrophages were recruited to the lung (Fig. 2a and Supplementary Fig. S3a) and abundant IL-1β was released (Figs. 2b and 5c), which indicates that Caspase-1-mediated pyroptosis in macrophages plays an important role in the development of RILI.

### Andrographolide inhibited radiation-induced AIM2 inflammasome-dependent pyroptosis in macrophages

As our work reported, Andrographolide suppressed nuclear factor (NF)-κB and mitogen-activated protein kinase (MAPK) activation markedly^24^ and it may act as a reactive oxygen species (ROS) scavenger as proposed in a lung model for ovalbumin-induced lung injury^34^. So these pathways were detected in sections or lysis of lung tissue. We found that Andrographolide suppressed radiation-induced NF-κB and MAPK activation significantly (Supplementary Fig. S5a–c), but the inhibitory effects of Andrographolide still existed after incubation with ROS scavenger *N*-acetyl-*L*-cysteine and NADPH oxidase inhibitor Diphenyleneiodonium, which indicated that Andrographolide works in an ROS-independent manner (Supplementary Fig. S5d, e).

**Fig. 6:**
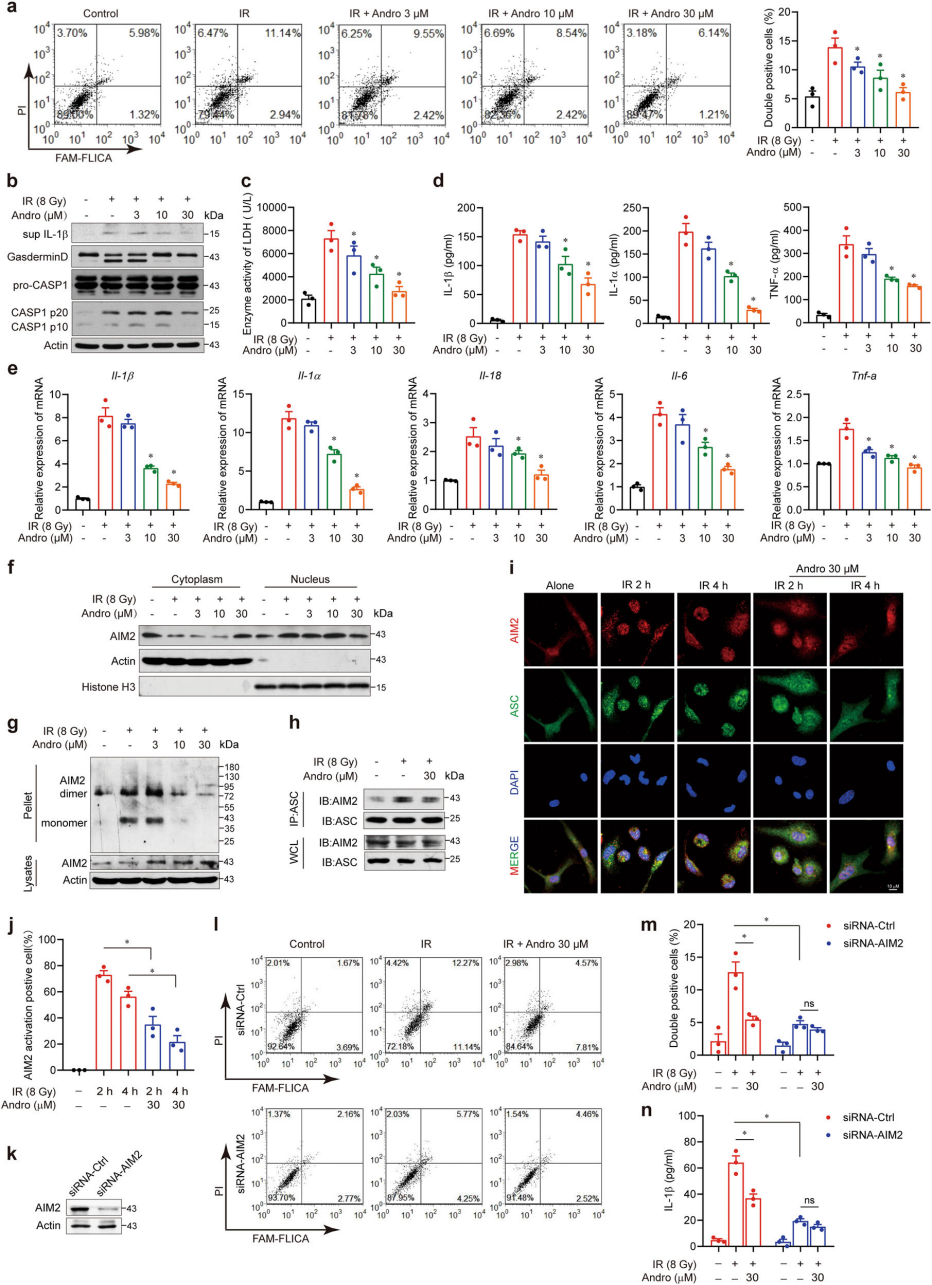
Andrographolide suppressed radiation-induced AIM2 inflammasome activation and pyroptosis in macrophages. Primarily cultured BMDMs were exposed to 8 Gy irradiation after incubation with or without the indicated concentrations of Andrographolide for 2 h. **a** Double positivity of Caspase-1 and PI were detected via flow cytometry after 24 h. **b** Relative protein levels of Gasdermin D and Caspase-1 were analyzed by western blot. **c** LDH activity in the supernatant was assessed by an LDH assay kit. **d** IL-1β, IL-1α, and TNF-α in the supernatant were detected by ELISA. **e** Relative mRNA expression of IL-1β, IL-1α, IL-18, IL-6, and TNF-α was examined by qPCR. **f** Protein levels of AIM2 in the cytosol and nucleus were assayed by western blot analyses. β-Actin and Histone H3 are shown as loading controls. **g** Cell lysates were separated by nonreducing SDS-PAGE and detected with antibodies against AIM2. **h** Co-immunoprecipitation of ASC with AIM2 was performed. The immunoprecipitates or whole-cell lysates were analyzed by immunoblotting with antibodies against AIM2 or ASC. **i** The subcellular localization of AIM2 (shown in red) and ASC (shown in green) was analyzed via confocal microscopy. Cell nuclei were visualized by DAPI (blue). Scale bar, 10 μm. **j** Fluorescence of co-localization of AIM2 and ASC was quantified (five fields per mouse in every group). **k**–**n** Effect of AIM2 silencing on Andrographolide-suppressed pyroptosis and IL-1β production in THP-1-derived macrophage. **k** AIM2 knockdown efficiency was detected by immunoblotting. **l**, **m** Double positivity of Caspase-1 and PI was detected by flow cytometry. **n** IL-1β level was detected by ELISA. The data shown are representative of three independent experiments (**a**, **b**, **f**, **g**, **h**, **i**, **k**, **l**). Data are expressed as the mean ± S.E.M. of three independent experiments (**c**, **d**, **e**, **j**, **m**, **n**). *P < 0.05 vs the IR group.

To address the underlying mechanism, we exposed BMDMs to 8 Gy of radiation and detected activated Caspase-1 and PI after 24 h. The proportion of double-positive BMDM was significantly increased by radiation, implying a Caspase-1-dependent cell death in macrophages (Fig. 6a). In addition, Gasdermin D, which is essential for pyroptosis, was cleaved by active Caspase-1 (Fig. 6b). The production of LDH, IL-1β (Fig. 6b), IL-1α, and TNF-α in the supernatant and the mRNA levels of IL-1β, IL-1α, IL-18, IL-6, and TNF-α were also markedly elevated (Fig. 6c–e). However, BMDMs treated with Andrographolide were resistant to radiation. Activation of the AIM2 inflammasome induces pyroptosis in macrophages. Upon binding to dsDNA via its HIN200 domain, AIM2 recruits the adapter protein ASC and assembles into an inflammasome to activate Caspase-1, resulting in maturation and secretion of IL-1β and IL-18^12,35^. In our study, we found that Andrographolide suppressed the activation and assembly of the AIM2 inflammasome in macrophages, as shown by the inhibitory effects on the translocation from the cytoplasm to nucleus in response to radiation (Fig. 6f), AIM2 oligomerization (Fig. 6g), and the interaction between ASC and AIM2 (Fig. 6h). To uncover the cellular mechanism, we detected the distribution of AIM2 during radiation by immunofluorescence. Endogenous AIM2 showed very diffuse and weak staining in resting macrophages but formed puncta in the nucleus at 2 h post-radiation, suggesting that AIM2 is recruited to the sites of DSBs. Upon radiation exposure for 4 h, AIM2 accumulated in the perinuclear region and co-localized with ASC (shown in yellow specks). Notably, Andrographolide treatment prevented AIM2 from migrating into the sites of DSBs in the nucleus and abrogated AIM2–ASC oligomerization (Fig. 6i, j). In addition, etoposide (VP16) is a commonly used chemotherapeutic drug in non-small cell lung cancer that can cause DNA damage to activate the AIM2 inflammasome. We found that VP16-induced LDH and IL-1β release, Caspase-1 activation, and assembly of the AIM2 inflammasome were suppressed by Andrographolide in macrophages (Supplementary Fig. S6a–d). We also observed that recruitment of AIM2 to the sites of DSBs, shown by nuclear co-localization of AIM2 and p-Histone-H2A.X, a sensitive marker for DNA damage, was blocked by Andrographolide treatment (Supplementary Fig. S6e). To confirm that Andrographolide is working in an AIM2-dependent manner, we performed experiments using AIM2 siRNA in THP-1-derived macrophage and BMDMs from NLRP3 knockout (KO) mice (Fig. 6 and Supplementary Fig. S7). The inhibitory effects of Andrographolide on radiation-induced pyroptosis and IL-1β secretion were still existing in BMDMs from NLRP3 KO mice (Supplementary Fig. S7), suggesting that NLRP3 deletion cannot attenuate the effect of Andrographolide on radiation-induced cell death and inflammasome activation. However, the inhibitory effects of Andrographolide on radiation-triggered pyroptosis and IL-1β production were almost completely blocked in THP-1-derived macrophages when AIM2 was silenced (Fig. 6l–n), indicating that the suppressive action of Andrographolide on radiation-induced cell death is dependent on AIM2. Thus Andrographolide significantly blocked radiation- or VP16-induced pyroptosis in BMDMs.

**Fig. 7:**
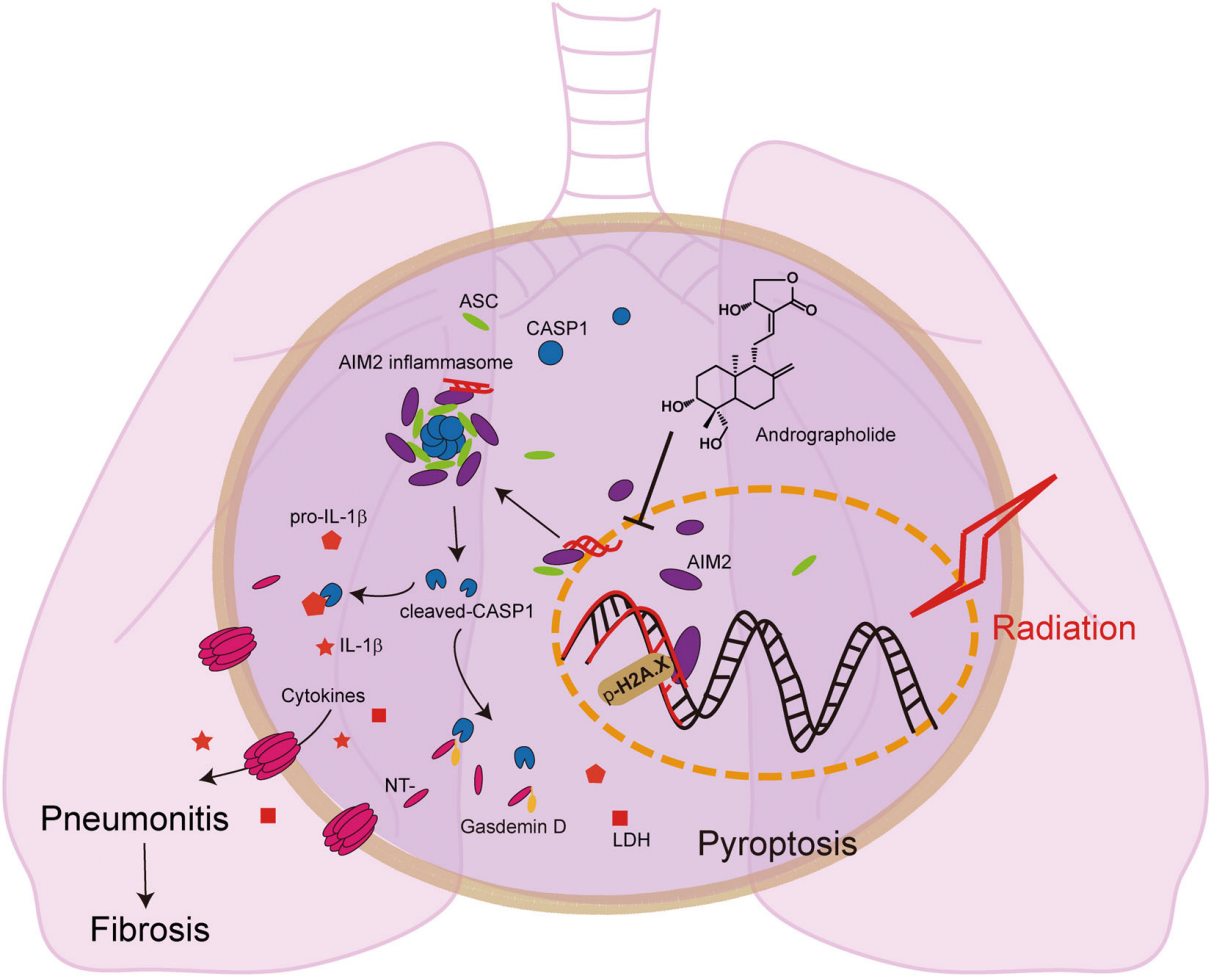
A graphic illustration of the mechanism of Andrographolide ameliorating radiation-induced lung injury. Andrographolide inhibits Caspase-1-mediated Gasdermin D-dependent pyroptosis in macrophage by preventing AIM2 from translocating into nucleus to sense DNA damage induced by radiation, thus alleviating radiation-induced lung inflammation and fibrosis.

Taken together, Andrographolide inhibits Caspase-1-mediated Gasdermin D-dependent pyroptosis in macrophage by preventing AIM2 from translocating into nucleus to sense DNA damage induced by radiation, thus alleviating radiation-induced lung inflammation and fibrosis (Fig. 7).

## Discussion

Radiotherapy has been regarded as a vital treatment for >70% of thoracic tumors^36^. However, the lung is relatively sensitive to irradiation^37^, and approximately 13–37% of patients who received radiotherapy are susceptible to RILI^38,39^. At present, there are no therapeutic agents that can be used for RILI, and the underlying mechanism is controversial^40,41^. In this study, we demonstrated that 18 Gy of radiation led to pulmonary edema; congestion; and progressive thickened alveolar walls, collapsed alveoli, and collagen deposition, eventually resulting in respiratory failure in mice. Against this, Andrographolide effectively mitigated acute pneumonitis and pulmonary fibrosis in late periods of RILI and protected mice as shown by the prolonged survival and extinguished lung inflammation.

Tissues exposed to irradiation increase cytokine production at an early stage, which in turn recruited more immune cells. Furthermore, the inflammatory cascade promoted fibroblast proliferation and collagen deposition^42^. Previous studies demonstrated that RILI could be alleviated by blocking pro-inflammatory cytokines, such as TNF-α, IL-1β, and IL-6, as well as the pro-fibrotic cytokine TGF-β1^43^. Consistent with these studies, Andrographolide significantly decreased the levels of IL-1β, IL-6, and TNF-α both in the plasma and in BALF. In addition, Andrographolide suppressed the accumulation of macrophages, neutrophils, and T lymphocytes in lung tissues. Furthermore, the mRNA expression of these cytokines was also apparently downregulated by Andrographolide as a result of blocked NF-κB signaling, which was reported in our previous study^24^.

Macrophages are recruited as a response to radiation exposure and were reported to play a key role in the pathogenesis of RILI^44–46^. Previous studies have demonstrated that radiation can result in pyroptosis in multiple organs and tissues, such as the intestinal epithelium^33^, liver, and muscles^47,48^. Nevertheless, whether pyroptosis in macrophages contributes to RILI remains unclear. Our results showed that radiation-induced cell death and Caspase-1 activation in lung tissue could be suppressed by Andrographolide treatment (Fig. 5). Based on these findings, we hypothesized that Andrographolide attenuated RILI by inhibiting Caspase-1-mediated cell death. Then we exposed BMDM to 8 Gy of radiation and detected pyroptosis by active Caspase-1 and PI double staining. Our current data confirmed that radiation exposure triggered pyroptosis in BMDMs (Fig. 6), which is further supported by the cleavage of Gasdermin D and the release of LDH, IL-1β, IL-1α, and TNF-α in the supernatant. As hypothesized, Andrographolide significantly blocked pyroptosis in BMDM induced by radiation. Although we provided the evidence for the contribution of pyroptosis to RILI, whether other forms of cell death, such as apoptosis, were also involved in this process requires further investigation.

AIM2 is an important inflammasome component that senses potentially dangerous cytoplasmic DNA, leading to activation of the ASC pyroptosome and Caspase-1, which further triggers the release of IL-1β^12^. Previously, AIM2-deficient mice were protected from subtotal body irradiation-induced lethality and intestinal damage^10^. However, the influence of radiation on the AIM2 inflammasome in RILI has not been reported. Herein, we found that radiation exposure resulted in AIM2 translocation into the nucleus to sense the damaged dsDNA, followed by AIM2 oligomerization and assembly and activation of the AIM2 inflammasome, while Andrographolide abrogated the AIM2 inflammasome-mediated pyroptosis in macrophages. Liu et al. reported that NLRP3 KO rescued a fraction of BMDMs from radiation-induced death and pyroptosis^9^. Other inflammasomes, such as NLRP1 and NLRC4, can recognize pathogen-associated molecular patterns that may also contribute to radiation-induced Caspase-1 activation^49,50^. Nevertheless, radiation can cause tissue damage via inflammasome activation, which in turn increases susceptibility to infections.

Above all, we concluded that Andrographolide significantly hampered the activation of the AIM2 inflammasome and pyroptosis in macrophage by preventing AIM2 from translocating into nucleus to sense DNA damage induced by radiation. As a result, the inflammatory cascade, acute pneumonitis, and subsequent chronic fibrosis were attenuated. This study demonstrates the radioprotective effects of Andrographolide on RILI and provides insights into the underlying molecular mechanism of RILI.

## Supplementary information

Supplementary Figure S1

Supplementary Figure S2

Supplementary Figure S3

Supplementary Figure S4

Supplementary Figure S5

Supplementary Figure S6

Supplementary Figure S7

Supplementary Figure legends

Supplementary Table 1
