# Supplementary figures and images for: Inhibition of AIM2 inflammasome-mediated pyroptosis by Andrographolide contributes to amelioration of radiation-induced lung inflammation and fibrosis
Source: Cell Death Dis. 2019 Dec 20;10(12):957. doi: 10.1038/s41419-019-2195-8 (PMC6925222; doi:10.1038/s41419-019-2195-8)

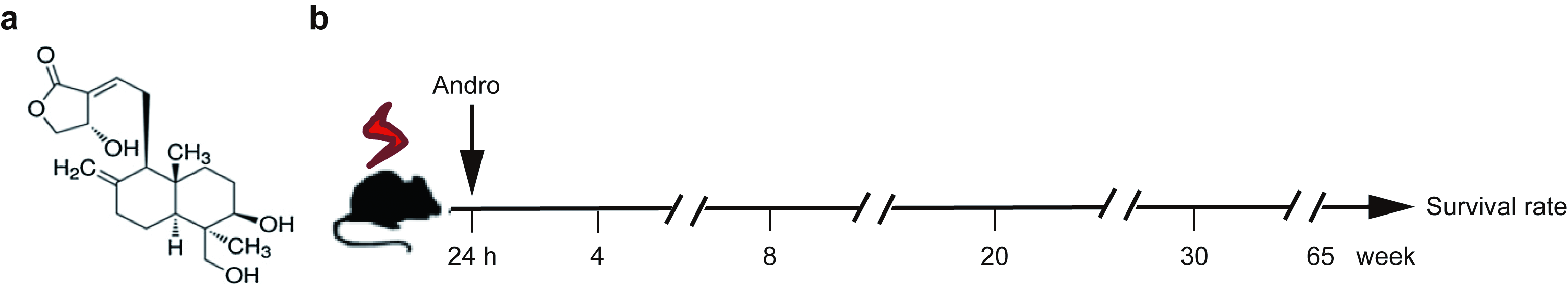

Supplement: Supplementary file 1 — Supplementary Figure S1 [file 41419_2019_2195_MOESM1_ESM.tif]

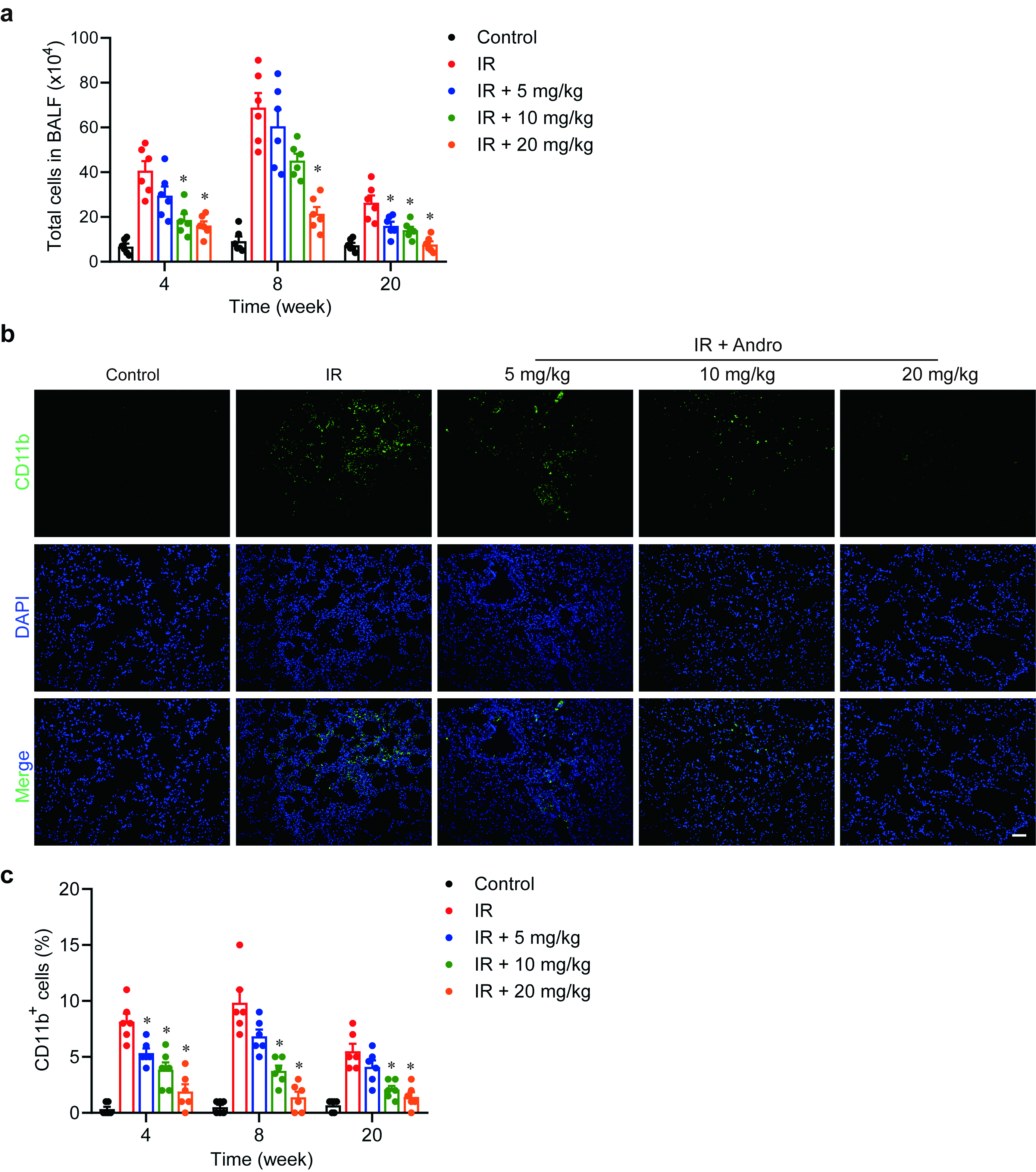

Supplement: Supplementary file 2 — Supplementary Figure S2 [file 41419_2019_2195_MOESM2_ESM.tif]

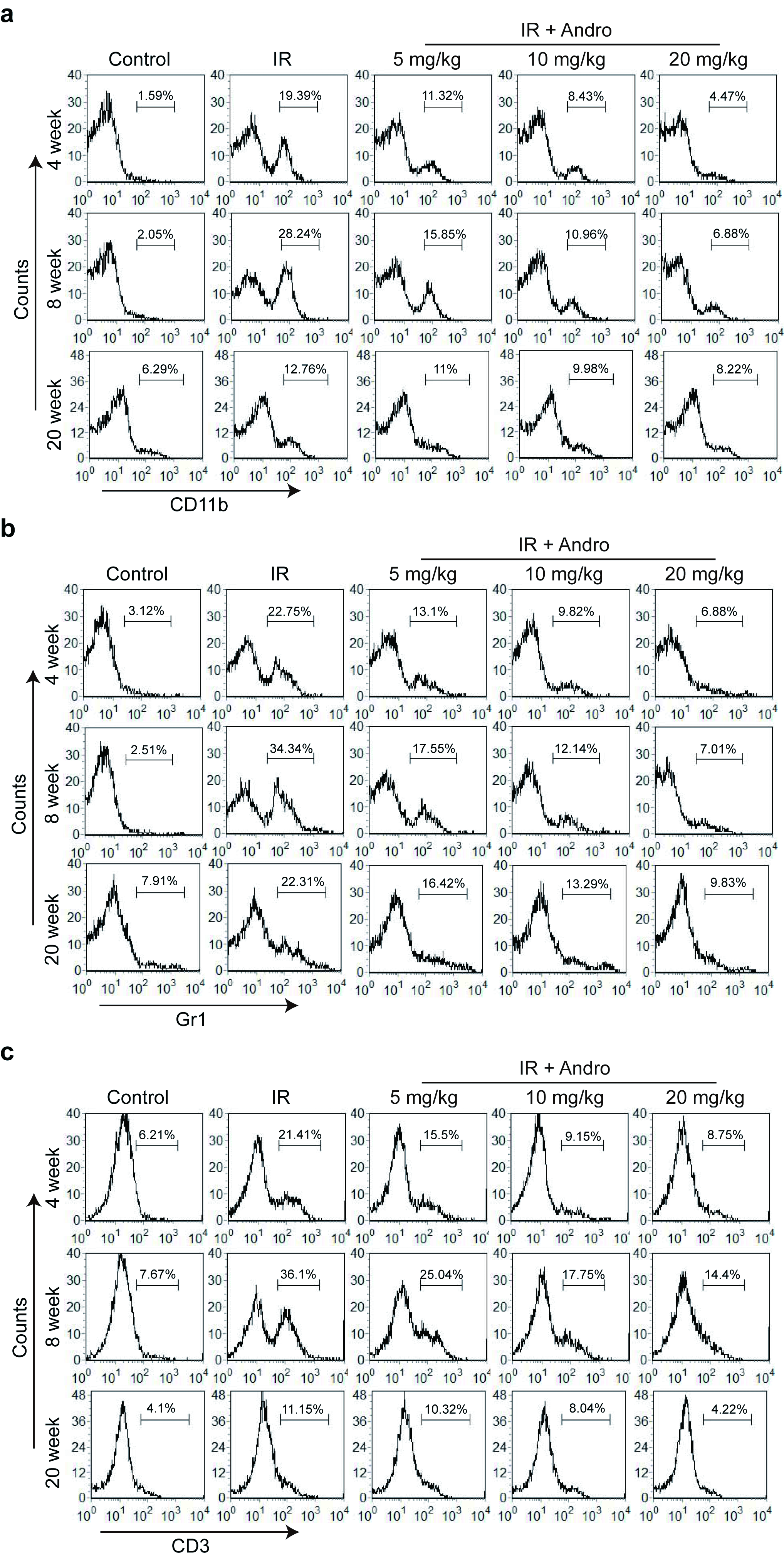

Supplement: Supplementary file 3 — Supplementary Figure S3 [file 41419_2019_2195_MOESM3_ESM.tif]

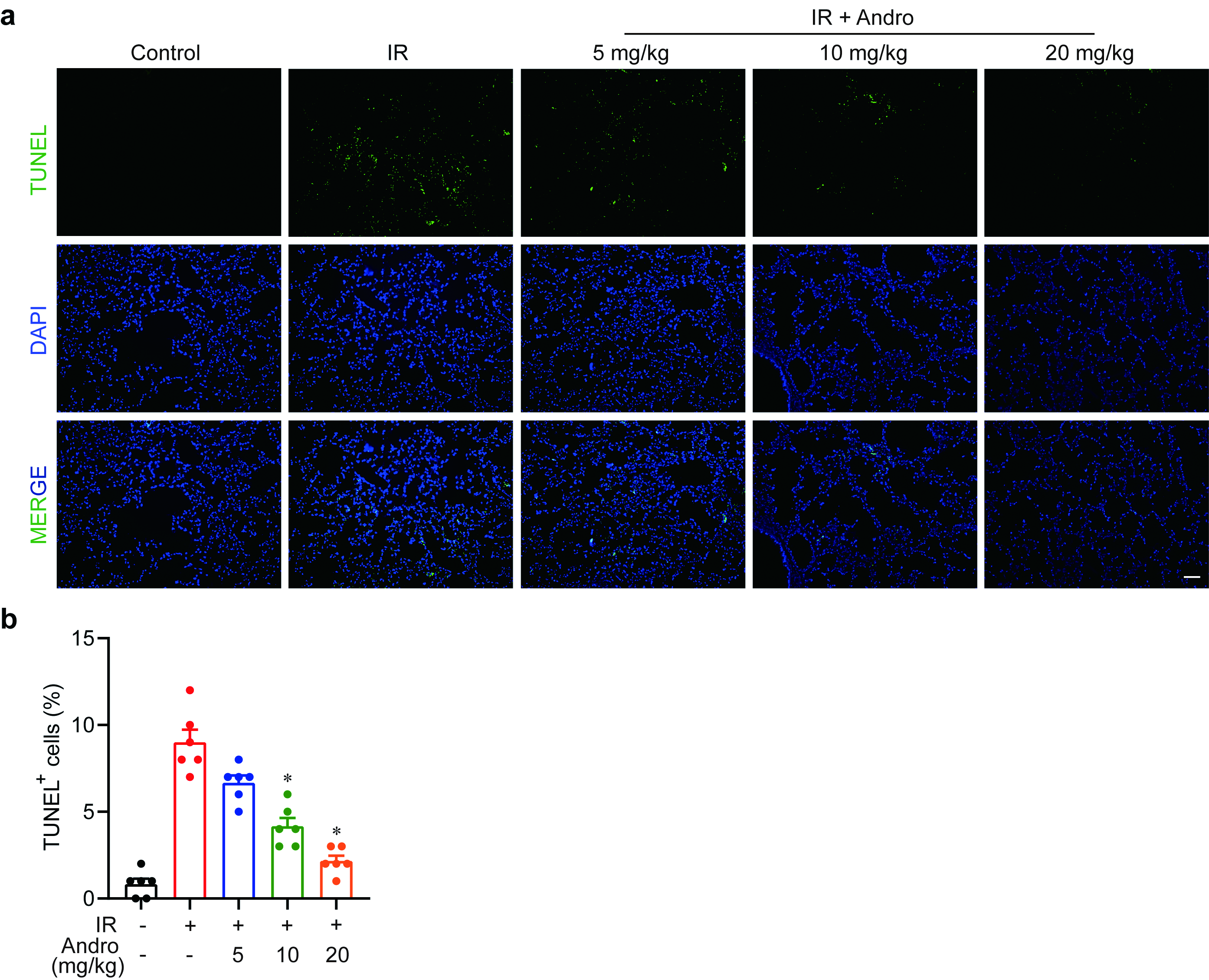

Supplement: Supplementary file 4 — Supplementary Figure S4 [file 41419_2019_2195_MOESM4_ESM.tif]

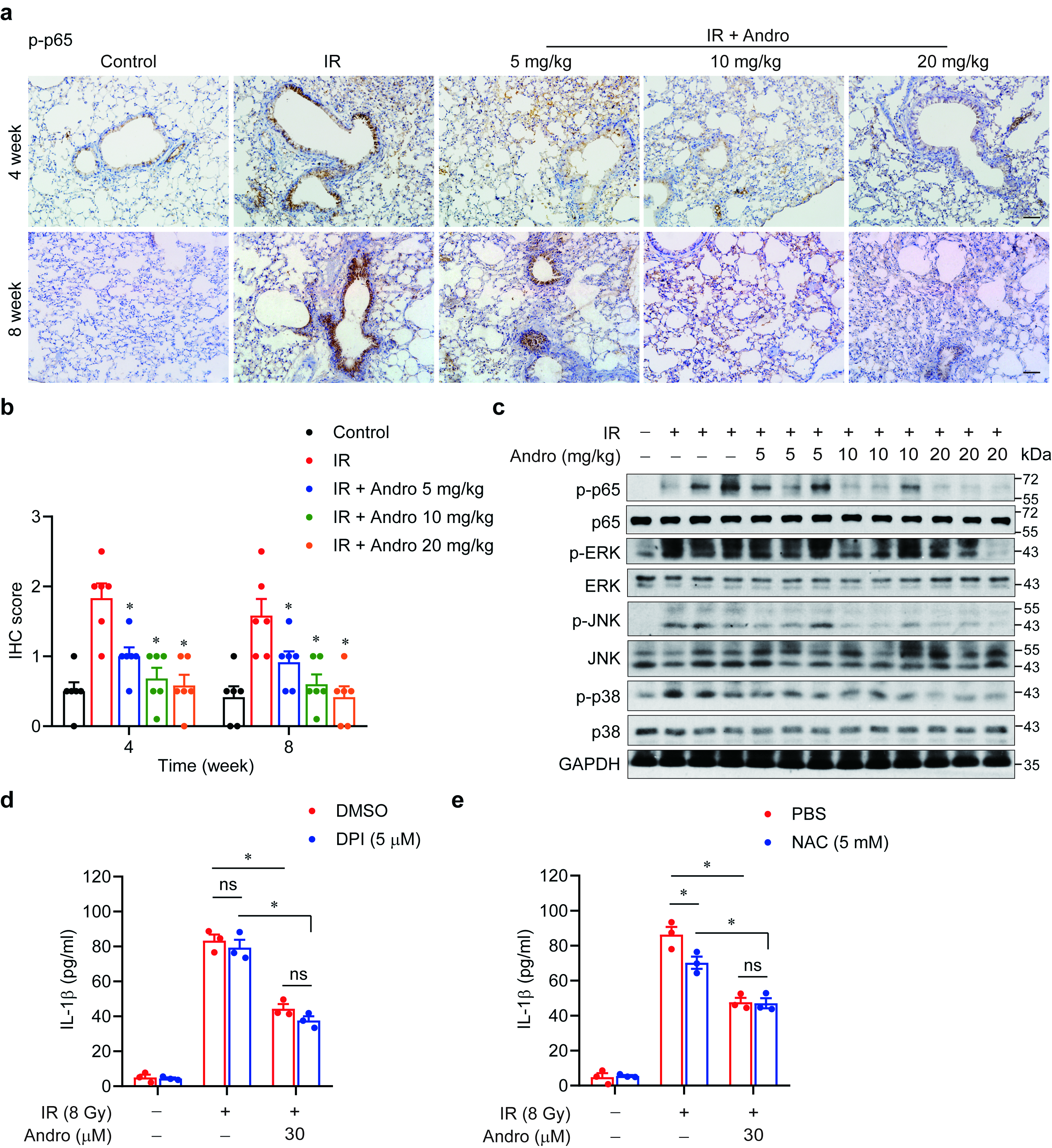

Supplement: Supplementary file 5 — Supplementary Figure S5 [file 41419_2019_2195_MOESM5_ESM.tif]

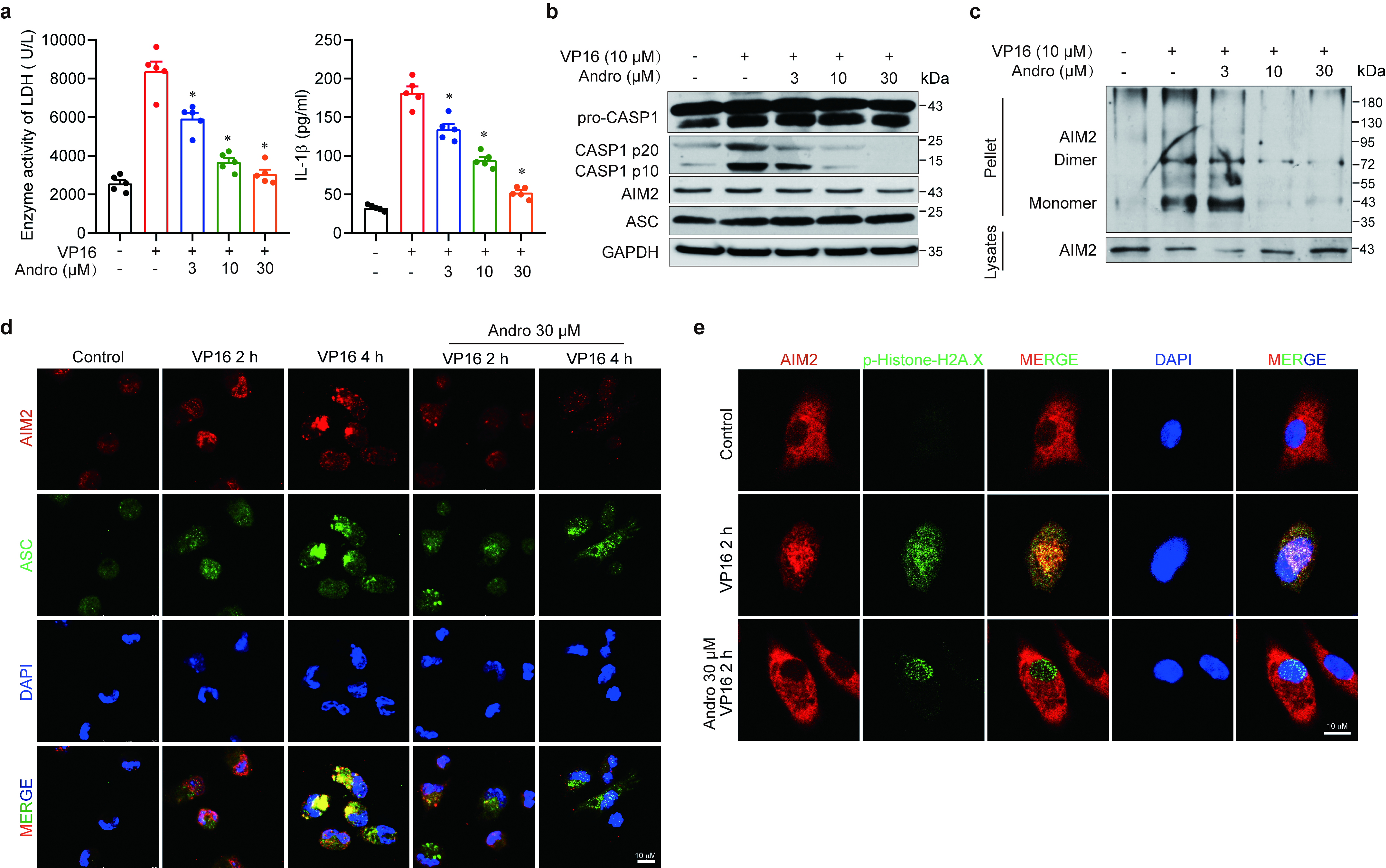

Supplement: Supplementary file 6 — Supplementary Figure S6 [file 41419_2019_2195_MOESM6_ESM.tif]

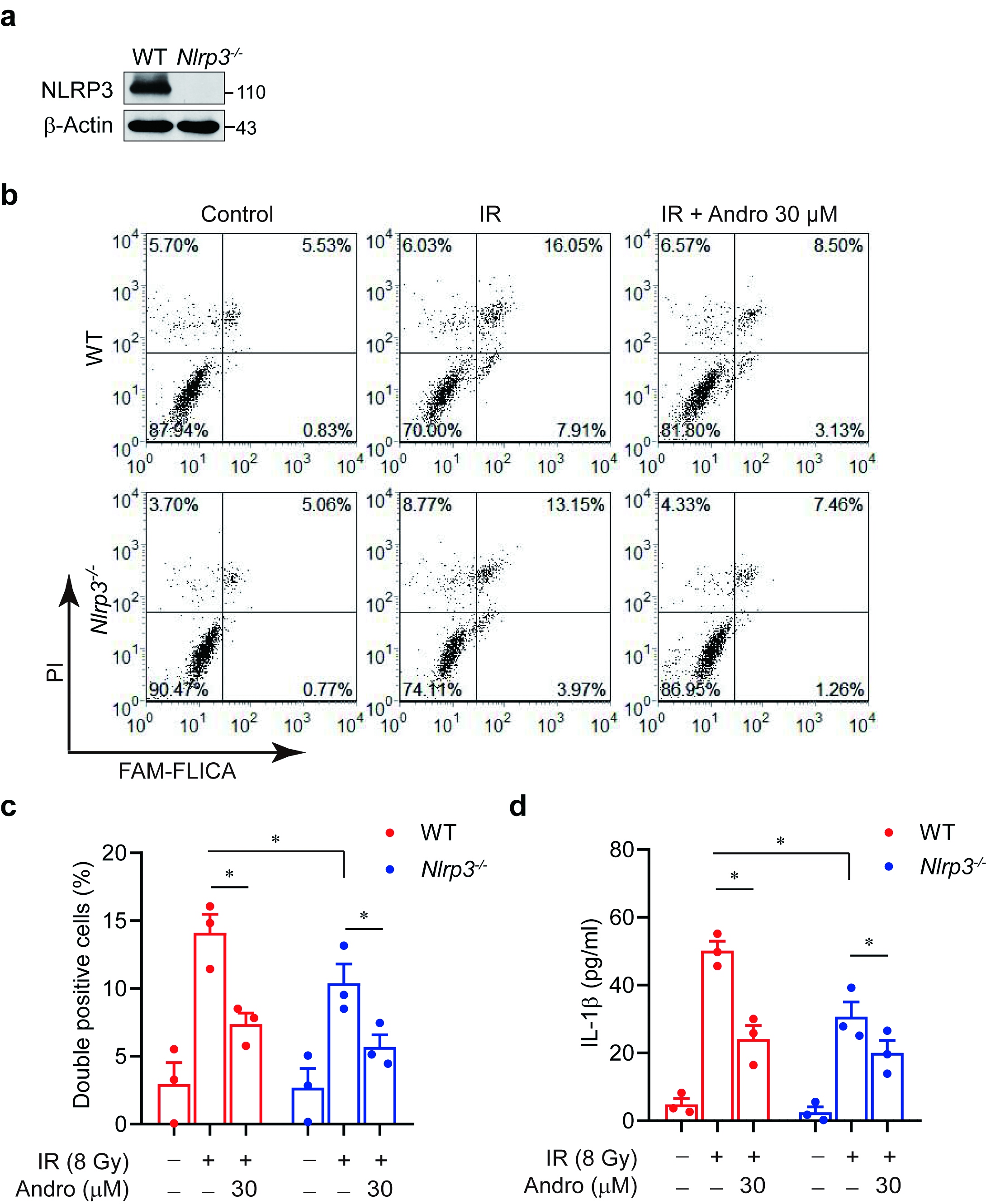

Supplement: Supplementary file 7 — Supplementary Figure S7 [file 41419_2019_2195_MOESM7_ESM.tif]
