# Supplementary material for: Inhibition of AIM2 inflammasome-mediated pyroptosis by Andrographolide contributes to amelioration of radiation-induced lung inflammation and fibrosis
Source: Cell Death Dis. 2019 Dec 20;10(12):957. doi: 10.1038/s41419-019-2195-8 (PMC6925222; doi:10.1038/s41419-019-2195-8)
Supplement: Supplementary file 8 — Supplementary Figure legends [file 41419_2019_2195_MOESM8_ESM.doc]

**Supplementary Figure legends**

**Supplementary Figure S1. Experimental design.** (**a**) Chemical structure of Andrographolide. (**b**) A schematic diagram of the experimental protocol.

**Supplementary Figure S2. Andrographolide diminished radiation-induced inflammatory cell infiltration.** (**a**) BALF from each group was collected and the total number of cells in BALF was counted. (**b**) Representative images of CD11b staining (shown in green). Nuclei were stained with DAPI (blue). Scale bar, 50 μm. (**c**) Numbers of CD11b-positive cells were quantified of five fields for per mouse in every group. Data are shown as the mean±S.E.M, n=6, **P*<0.05 vs the IR group.

**Supplementary Figure S3. Andrographolide attenuated inflammatory cell infiltration in the lung.** Andrographolide administration significantly attenuated radiation-induced lung infiltration of immune cells, shown as CD11b^+^ macrophages (**a**), Gr1^+^ neutrophils (**b**) and CD3^+^ T lymphocytes (**c**). Representative data are shown from per mouse in every group. n=6 per group.

**Supplementary Figure S4. Andrographolide attenuated radiation-induced cell death in lung tissues.** (**a**) Cell death was analyzed by TUNEL staining. Cells stained positively for TUNEL showed green fluorescence. Nuclei were stained with DAPI (blue). Scale bar, 50 μm. (**b**) The numbers of TUNEL positive cells was quantified of five fields for per mouse in every group. Data are shown as the mean±S.E.M, n=6, **P*<0.05 vs the IR group.

**Supplementary Figure S5. Andrographolide inhibited radiation-induced NF-κB and MAPKs activation in lung tissues in a ROS-independent manner.** (**a**) Representative images of immunohistochemistry staining for p-p65. Scale bar, 50 μm. (**b**) IHC score of p-p65. Data are shown as the means±S.E.M of five fields of view per mouse in every group, n=6 mice per group. **P*<0.05 vs the IR group. (**c**) Western blot analysis of NF-κB and MAPKs in lung tissues from different groups at 4 week after irradiation. (**d, e**) Primarily cultured BMDMs were exposed to 8 Gy irradiation after incubation with or without Andrographolide (30 μM) and DPI (5 μM)/NAC (5 mM). IL-1β in the supernatant was detected by ELISA after 24 h. The data shown are representative of three independent experiments (**a, c**). Data are expressed as the mean±S.E.M of three independent experiments (**d, e**). **P*<0.05.

**Supplementary Figure S6. Andrographolide inhibited VP16-induced AIM2 inflammasome activation in macrophages.** Primarily cultured BMDMs were stimulated with 10 μM VP16 in the presence or absence of Andrographolide. (**a**) LDH activity in the supernatant was assessed by an LDH Cytotoxicity Assay Kit. IL-1β in the supernatant was detected by ELISAs. Data represent the mean±S.E.M of five independent experiments. **P*<0.05 vs the VP16 group. (**b**) Immunoblot analysis of AIM2 inflammasome-related proteins. (**c**) Cell lysates were separated by nonreducing SDS-PAGE and detected with antibodies against AIM2. (**d**) The subcellular localization of AIM2 (shown in red) and ASC (shown in green) was analyzed via confocal microscopy. Cell nuclei were visualized by DAPI (blue). Scale bar, 10 μm. (**e**) Fluorescence of co-localization of AIM2 (shown in red) and p-Histone-H2A.X (shown in green) was analyzed via confocal microscopy. Cell nuclei were visualized by DAPI (blue). Scale bar, 10 μm. The data shown are representative of three independent experiments (**b, c, d, e**).

**Supplementary Figure S7. NLRP3 deficiency cannot attenuate the inhibitory effect of Andrographolide on radiation-induced cell death.** (**a**) NLRP3 protein levels from primarily cultured BMDMs of WT and *Nlpr3^-/-^* mice were analyzed by Western blot. BMDMs were exposed to 8 Gy irradiation followed by incubation with or without the indicated concentrations of Andrographolide. Double positivity of Caspase-1 and PI were detected via flow cytometry after 6 h (**b, c**). IL-1β in the supernatant was detected by ELISA (**d**). The data shown are representative of three independent experiments (**a, b**). Data are expressed as the mean±S.E.M of three independent experiments (**c, d**). **P*<0.05.
