# Supplementary material for: Inhibition of AIM2 inflammasome-mediated pyroptosis by Andrographolide contributes to amelioration of radiation-induced lung inflammation and fibrosis
Source: Cell Death Dis. 2019 Dec 20;10(12):957. doi: 10.1038/s41419-019-2195-8 (PMC6925222; doi:10.1038/s41419-019-2195-8)
Supplement: Supplementary file 9 — Supplementary Table 1 [file 41419_2019_2195_MOESM9_ESM.doc]

**Supplementary Table 1.** Primer sequences for qPCR.

| Primer | Forward | Reverse |
| --- | --- | --- |
| *Tnf-α* | 5’-CGAGTGACAAGCCTGTAGCCC-3’ | 5’-GTCTTTGAGATCCATGCCGTTG-3’ |
| *IL-1α* | 5’-CGAAGACTACAGTTCTGCCATT-3’ | 5’-GACGTTTCAGAGGTTCTCAGAG-3’ |
| *IL-1β* | 5’-CTTCAGGCAGGCAGTATCACTC-3’ | 5’-TGCAGTTGTCTAATGGGAACGT-3’ |
| *IL-6* | 5’-ACAACCACGGCCTTCCCTAC-3’ | 5’-TCTCATTTCCACGATTTCCCAG-3’ |
| *Tgfb* | 5’-AGACCACATCAGCATTGAGTG-3’ | 5’-GGTGGCAACGAATGTAGCTGT-3’ |
| *Cdh1* | 5’-AATGAAGCCCCCATCTTTAT-3’ | 5’-GCGTCTTCTCTGTCCATCTC-3’ |
| *Cdh2* | 5’-AGGCTTCTGGTGAAATTGCAT-3’ | 5’-GTCCACCTTGAAATCTGCTGG-3’ |
| *Col1a* | 5’-TCAGGTCACAGCCTTGATAAGC-3’ | 5’-GCAGATCTACTACTCAGACAAG-3’ |
| *Acta2* | 5’-ATTGTGCTGGACTCTGGAGATGGT-3’ | 5’-TGATGTCACGGACAATCTCACGCT-3’ |
| *Vim* | 5’-CGTCCACACGCACCTACAG-3’ | 5’-GGGGGATGAGGAATAGAGGCT-3’ |
| *β-Actin* | 5’-TGCTGTCCCTGTATGCCTCT-3’ | 5’-TTTGATGTCACGCACGATTT-3’ |
